# Supplementary material for: Macrosphelide A Exhibits a Specific Anti-Cancer Effect by Simultaneously Inactivating ENO1, ALDOA, and FH
Source: Pharmaceuticals (Basel). 2021 Oct 19;14(10):1060. doi: 10.3390/ph14101060 (PMC8541406; doi:10.3390/ph14101060)
Supplement: Supplementary file 1 [file pharmaceuticals-14-01060-s001.zip › pharmaceuticals-1307375-supplementary.pdf]

pharmaceuticals

MDPI

Macrosphelide A Exhibits a Specific Anti-Cancer Effect by Simultaneously Inactivating ENO1, ALDOA, and FH

Kyoung Song [1,†], Nirmal Rajasekaran [2,†], Chaithanya Chelakkot [3], Hunseok Lee [2], Seungmann Paek [4], Hobin Yang [2], Lina Jia [5], Heegeon Park [6], Woosung Son [7], Yujin Kim [8], Joonseok Choi [9], Haemin Jeong [10], Youngger Suh [7], Hwayoung Yun [11,*] and Youngkee Shin [2,3,6,*]

Supplementary Figures

Figure S1.

A

MSPA

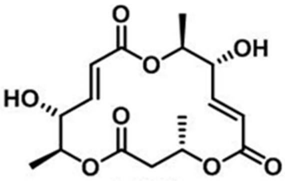

B

MSPA-biotin-735

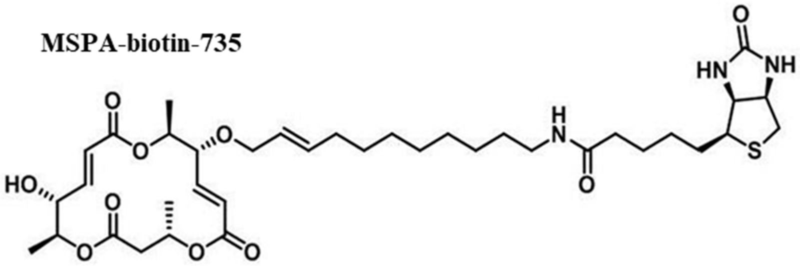

MSPA-biotin-961

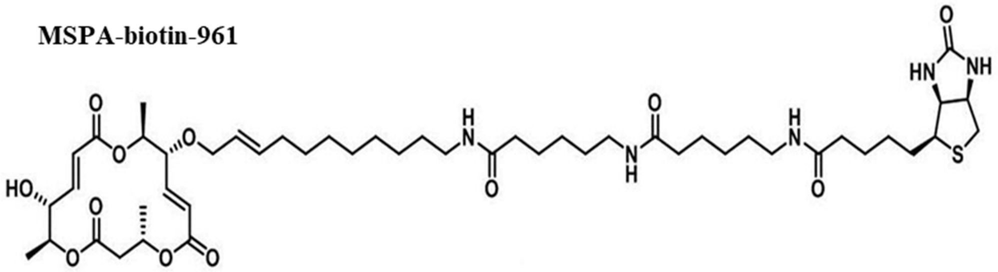

C

MSPB

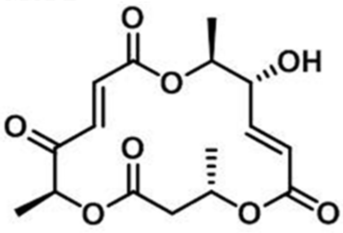

Butenyl

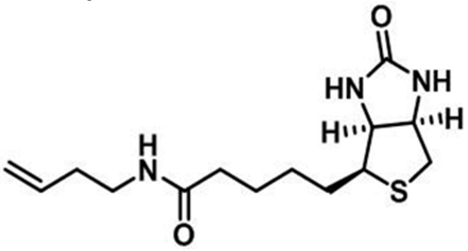

pharmaceuticals

MDPI

Figure S2.

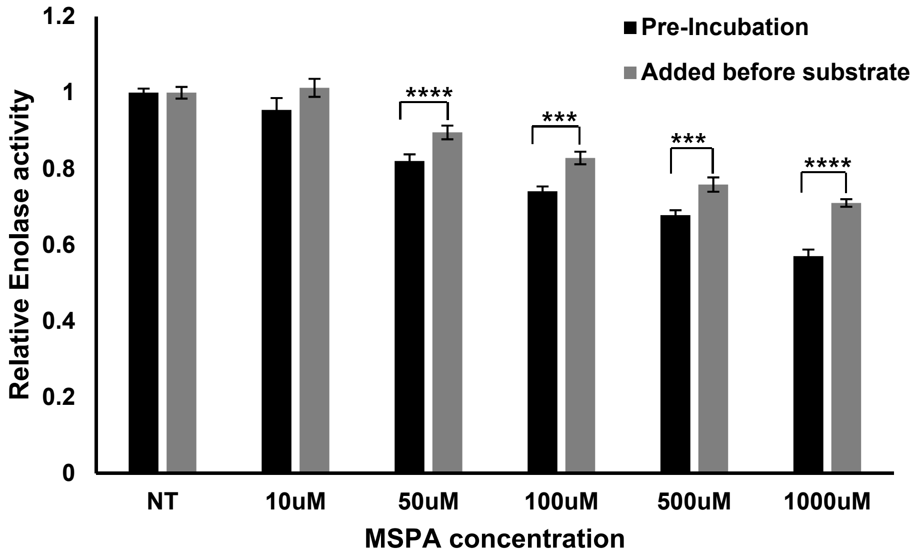

MSPA inhibits enolase enzyme activity. Measurement of the effect of MSPA on the enzymatic activity of purified *ENO1* by direct measurement of PEP at 240nm. Pre-incubation of MSPA showed significant inhibition at lower concentration of MSPA and also showed more significant inhibition than when the inhibitor was added just before adding the substrate. Data represent the mean ± SD (n = 3). *** $p < 0.001$, **** $p < 0.0001$ versus the control group.

pharmaceuticals

MDPI

Figure S3.

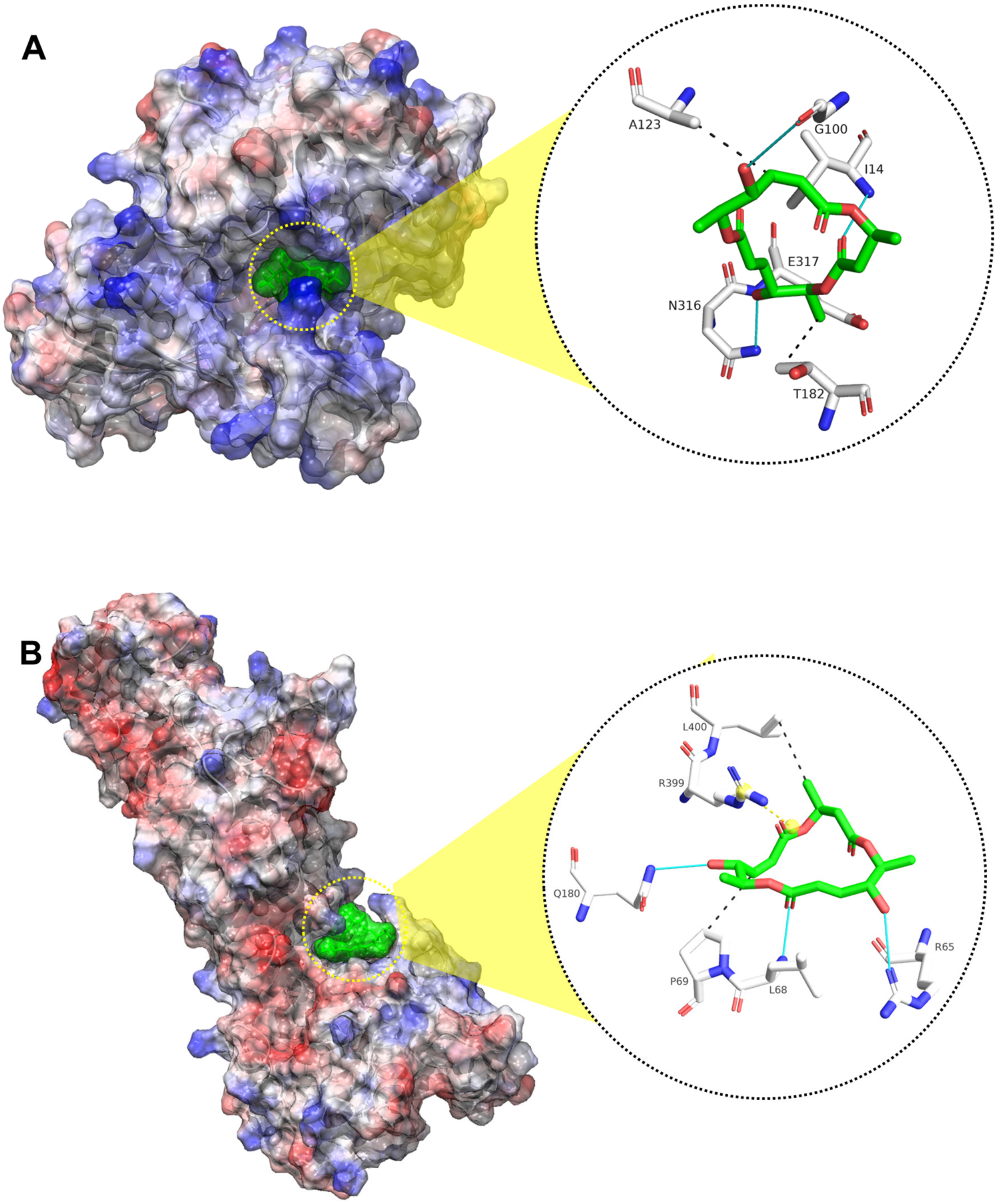

pharmaceuticals

C

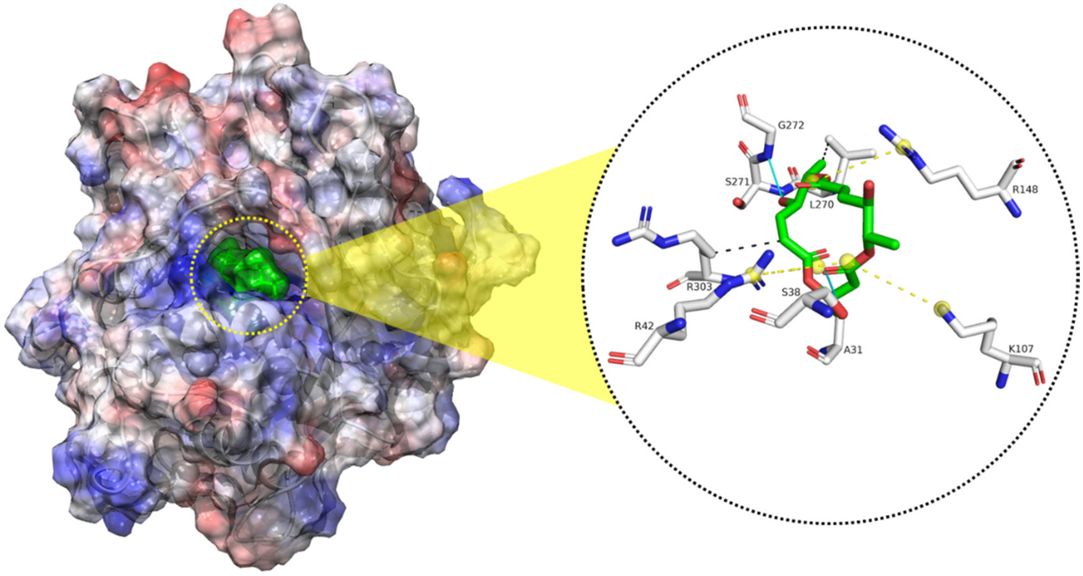

Molecular docking calculation of MSPA-protein complexes (A: GAPDH, B: FH, C:ALDOA). Complex models of four proteins were represented with electro surface potentials (ESP) and stick method. Positive charged surface region was colored as blue and negative charged surface region was red color, respectively. Heavy atoms were yellow (for sulfur), red (for oxygen), and blue (for nitrogen) in stick model of binding site. MSPA molecule was colored with green and interactions of proteins were represented with cyan lines (for hydrogen bond), yellow dots (for salt bridge), and gray dots (for hydrophobic interaction), respectively.

Figure S4.

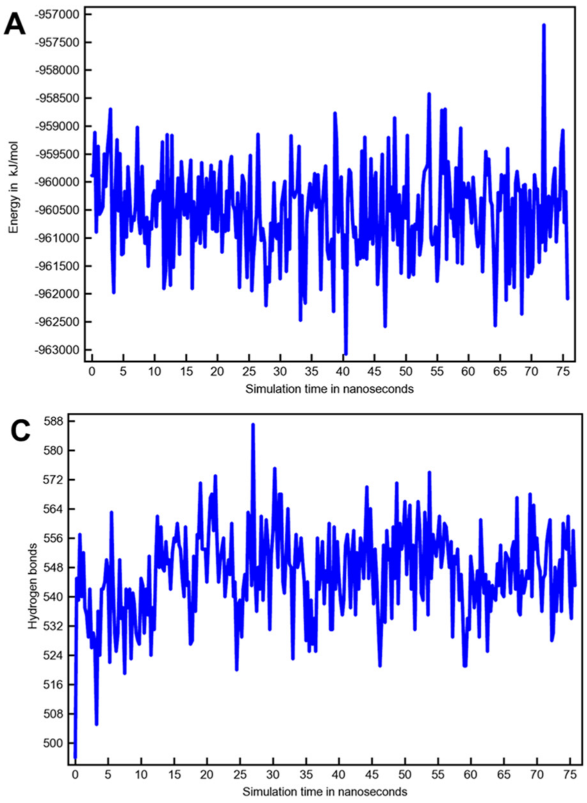

Molecular dynamics simulation of MSPA-GAPDH. The total potential energy of MSPA-GAPDH in AMBER14 force field was plotted in A. Numbers of hydrogen bonds in GAPDH (B) and between MSPA and GAPDH (C) were plotted and these numbers is a good indicator for proper structure folding. The RMSD plot of MSPA atoms over simulation time summarizing the conformational changes of the MSPA was represented in D. The whole simulation time length is about 75 nano seconds.

Figure S5.

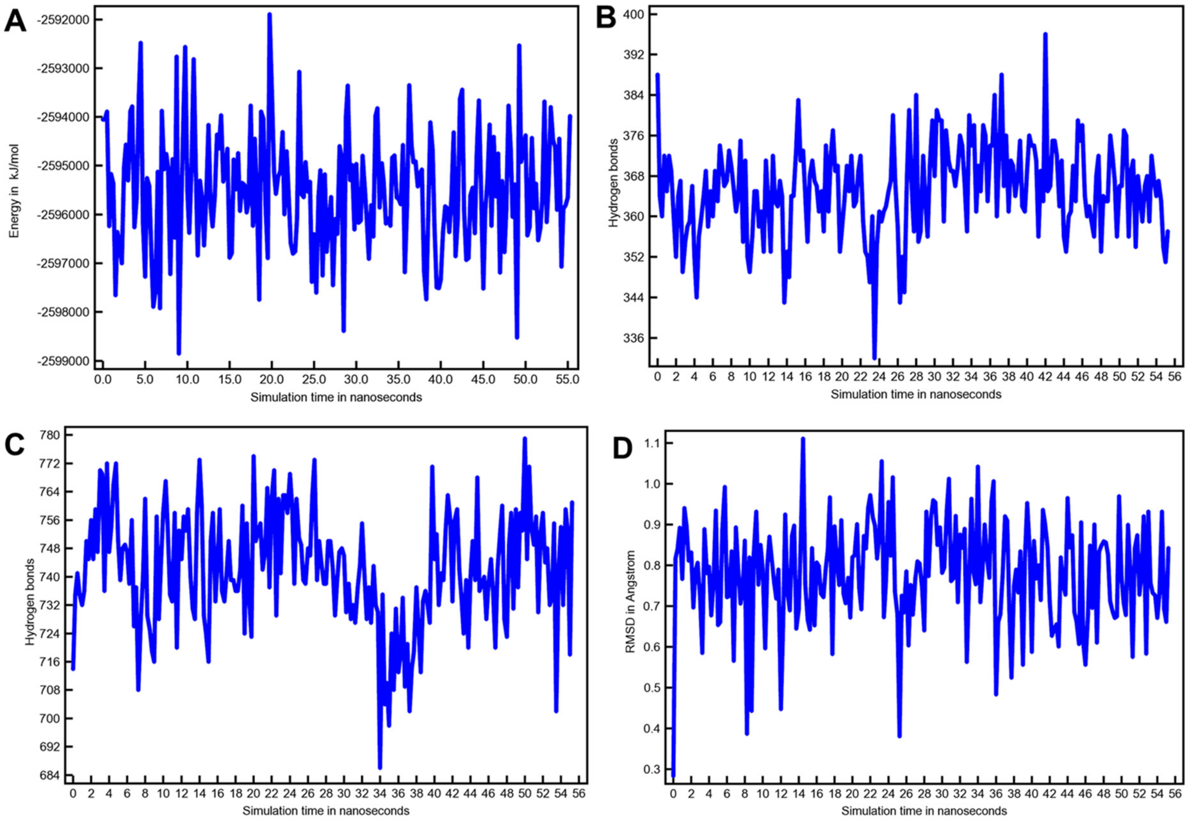

Molecular dynamics simulation of MSPA-FH. The total potential energy of MSPA-FH in AMBER14 force field was plotted in A. Numbers of hydrogen bonds in FH (B) and between MSPA and FH (C) were plotted and these numbers is a good indicator for proper structure folding. The RMSD plot of MSPA atoms over simulation time summarizing the conformational changes of the MSPA was represented in D. The whole simulation time length is about 56 nano seconds.

pharmaceuticals

Figure S6.

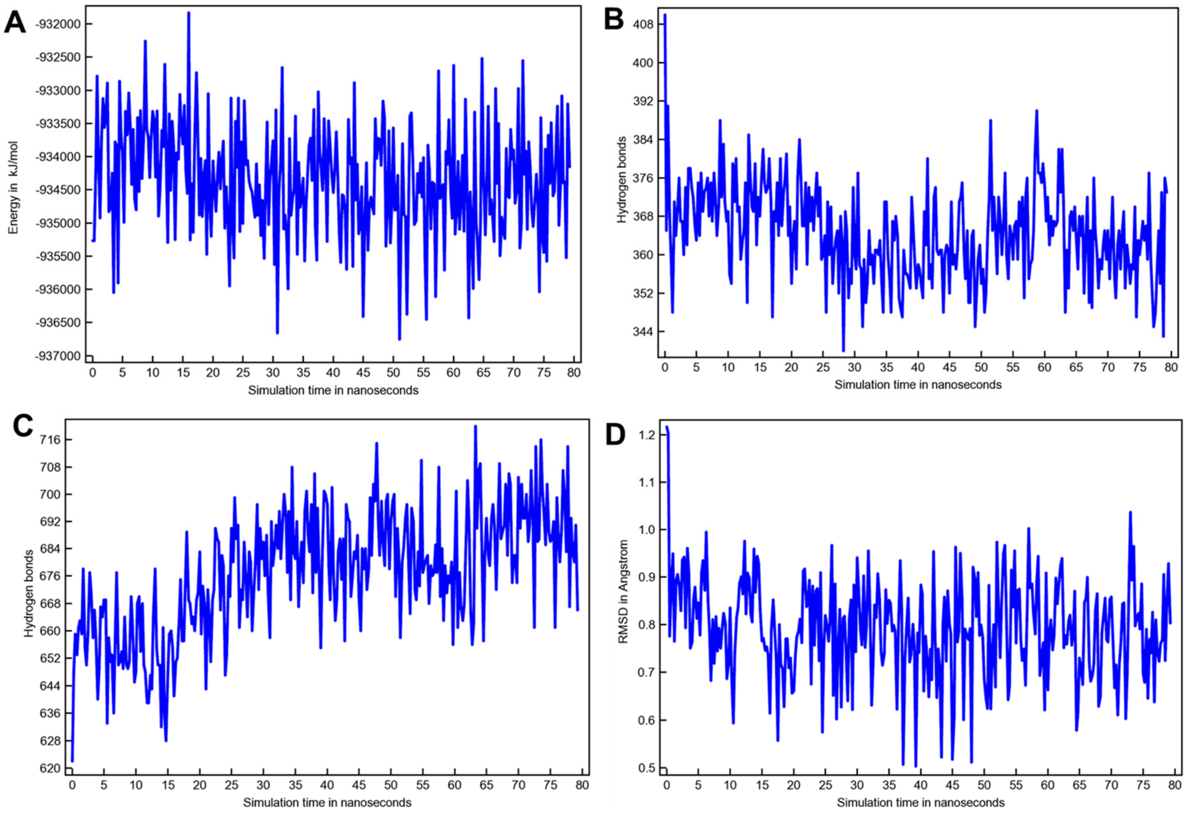

Molecular dynamics simulation of MSPA-ENO1. The total potential energy of MSPA-ENO1 in AMBER14 force field was plotted in A. Numbers of hydrogen bonds in ENO1 (B) and between MSPA and ENO1 (C) were plotted and these numbers is a good indicator for proper structure folding. The RMSD plot of MSPA atoms over simulation time summarizing the conformational changes of the MSPA was represented in D. The whole simulation time length is about 80 nano seconds.

Figure S7.

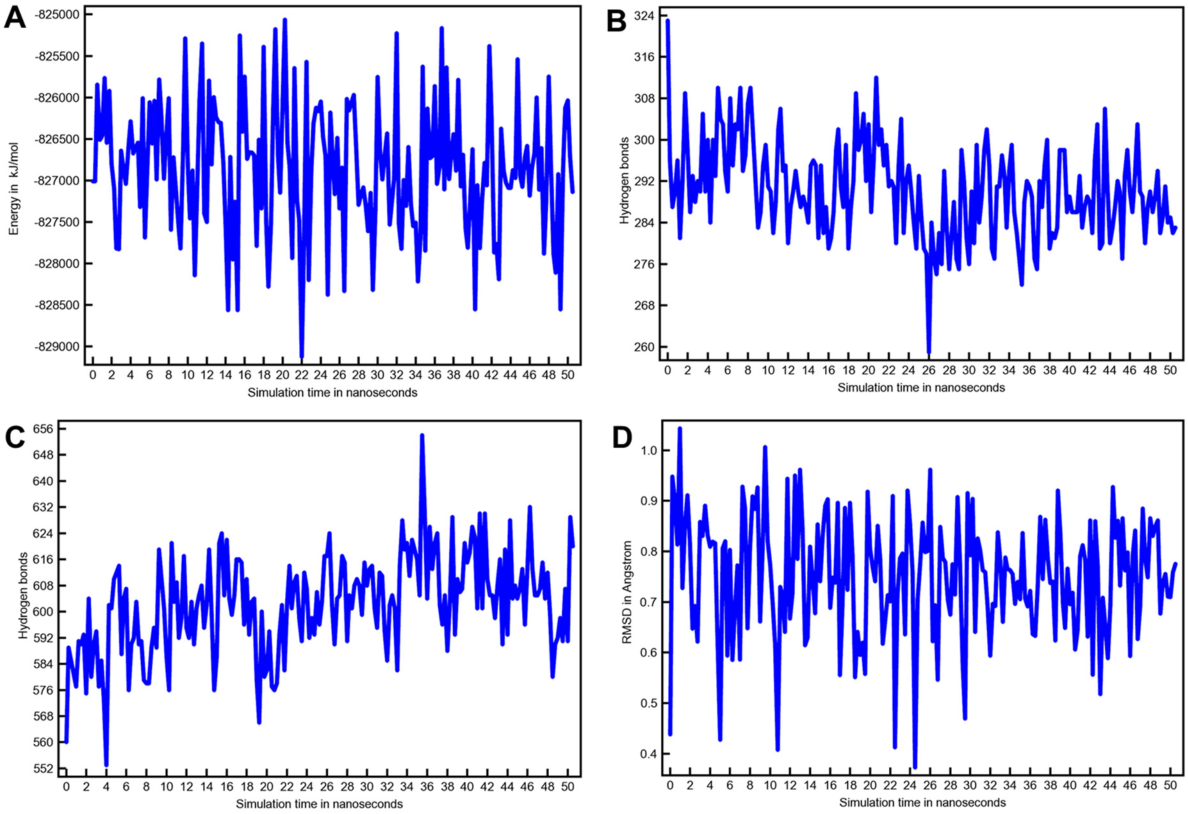

Molecular dynamics simulation of MSPA-ALDOA. The total potential energy of MSPA-ALDOA in AMBER14 force field was plotted in A. Numbers of hydrogen bonds in ALDOA (B) and between MSPA and ALDOA (C) were plotted and these numbers is a good indicator for proper structure folding. The RMSD plot of MSPA atoms over simulation time summarizing the conformational changes of the MSPA was represented in D. The whole simulation time length is about 50 nano seconds.

pharmaceuticals

MDPI

Supplementary Table S1. The primer and probe sequences for qRT-PCR

Gene	Primer Sequence (5′–3′)	UPL No.
ALDOA (S)	GGGAAGAAGGAGAACCTGAAG	# 63
ALDOA (AS)	TGACAGGCAAGGCTGTTG	
ENO1 (S)	GCTCCGGGACAATGATAAGA	# 60
ENO1 (AS)	TGATGTGCTCAACAGCCTTT	
GAPDH (S)	CCATGTTCGTCATGGGTGT	# 90
GAPDH (AS)	CCAGGGGTGCTAAGCAGTT	
FH (S)	TTGATCCAAAGATTGCTAATGC	# 74
FH (AS)	TGATCATTTAATTTACCTTCAGCTACC	
HPRT (S)	CTCAACTTTAACTGGAAAGAATGTC	
HPRT (AS)	TCCTTTTCACCAGCAAGCT	
	YAK	
HPRT (probe)	TTGCTTTCCTTGGTCAGGCAGTATAATC	
	BBQ	
